# MEDICAID MANAGED LONG-TERM SERVICES AND SUPPORTS: POLICY INNOVATION AND EVIDENCE GENERATION

**DOI:** 10.1093/geroni/igz038.2018

**Published:** 2019-11-08

**Authors:** Howard Degenholtz

**Affiliations:** University of Pittsburgh, Pittsburgh, Pennsylvania, United States

## Abstract

Twenty-two states have turned to managed care organizations to finance and deliver long-term services and supports (LTSS) as a way to control costs, improve quality and shift the locus of care away from institutional settings. These programs, referred to as Managed Long-Term Services and Supports (MLTSS) have been undertaken under several CMS authorities, and vary in terms of the program design and populations covered. Some programs such as the financial alignment demonstration, have integrated Medicaid and Medicare financing, while others have used waiver authority and three-party agreements to achieve coordination. However, all efforts share the same goal of improving the linkage between LTSS and both physical and behavioral health care. This presentation will provide conceptual framework for understanding and assessing program impact using the example of the multi-method evaluation of the $4 billion Pennsylvania Community HealthChoices program, one of the largest transitions in Medicaid policy to date.

